# A randomised controlled school-based nutritional intervention in five Middle Eastern countries: Ajyal Salima improved students’ dietary and physical activity habits

**DOI:** 10.1017/S1368980023001489

**Published:** 2023-10

**Authors:** Carla Habib-Mourad, Carla Maliha, Amira Kassis, Anh Thi Nguyen, Diala Ammar, Eman Haji, Lina AlTarazi, Suzanne Totah, Nahla Hwalla

**Affiliations:** 1 Department of Nutrition and Food Sciences, Faculty of Agriculture and Food Sciences, American University of Beirut, Riad El-Solh, Beirut 1107 2020, Lebanon; 2 Whiteboard Nutrition Science, 142 York Road, Beaconsfield, Canada; 3 The Association for Canadian Studies and Metropolis Institute, 50-1980 Sherbrooke, Street West Montreal, Quebec, Canada; 4 Department of health and Physical Education, Mount Royal University, 4825 Mount Royal Gate, Calgary, AB, Canada; 5 Ministry of Health, Building 929, Road 1015, Sanabis 410, Kingdom of Bahrain; 6 Royal Health Awareness Society, Muhammad As-Saeed Al-Batayni St., Amman 11821, Jordan; 7 Ministry of Education, Ramallah 00970, Palestine

**Keywords:** School-based intervention, Physical activity, Middle East, Childhood obesity, Nutrition education

## Abstract

**Objective:**

The purpose of this study was to measure the impact of the Ajyal Salima school intervention on nutrition and physical activity outcomes in children aged 9–11 years.

**Design:**

The study was a 1-year cluster-randomised controlled trial. Ajyal Salima used a multi-component approach including classroom activities, family programme and food service adaptation. Outcomes included daily intake of breakfast, frequency of healthy and unhealthy food consumption, frequency of physical activity, knowledge score and self-efficacy score. Intervention and control groups were compared for all main outcomes and a post-intervention qualitative evaluation assessed strengths and limitations of the intervention components.

**Setting:**

Schools in five countries – Lebanon, Jordan, Palestine, Saudi Arabia and Bahrain.

**Participants:**

Schools were selected by Ministries of Health and Education within their jurisdictions. Forty-five intervention schools (6052 students) and forty-six control schools (6200 students) were included in the analysis.

**Results:**

The intervention group had a significantly higher odds of consuming breakfast daily (OR 95 % CI = 1·60, 1·35, 1·90), consuming healthy foods (OR 95 % CI = 1·60, 1·39, 1·84) and a decreased odds of consuming unhealthy foods and sweetened beverages (OR, 95 % CI = 0·70, 0·60, 0·81). Additionally, school children in the intervention group, as compared with the control group, were 47 % more likely to exercise outside school hours (OR 95 % CI = 1·47, 1·23, 1·76). Lastly, children in the intervention group had a significantly improved nutritional knowledge score and improved self-efficacy by 1·3 score unit and 1·1 score unit, respectively.

**Conclusions:**

The Ajyal Salima intervention led to significant improvements in dietary and physical activity habits among school children and increased nutritional knowledge scores.

## Graphical abstract

**Figure:**
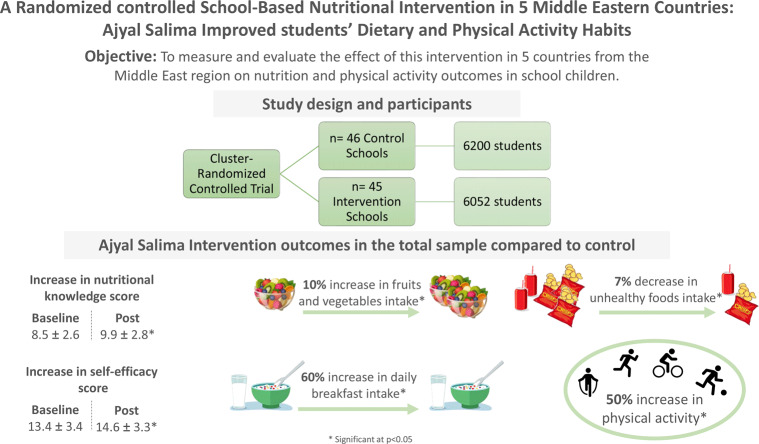

Globally, childhood obesity is a critical public health concern with concrete repercussions on metabolic health later in life. Overweight children in kindergarten were shown to be four times more likely than healthy-weight children to be overweight or obese at fourteen^(1)^. The global obesity trend is increasingly observed in the Middle Eastern region which has one of the highest rates of obesity worldwide^(2,3)^. This rising obesity trend is mirrored by a decrease in the quality of dietary patterns globally, and in the Eastern Mediterranean region in particular^(4)^. Recent research surveys show that children consume less fruits and vegetables, more energy-dense foods and an inadequately high intake of foods with low nutritional value^(5–10)^. Such unhealthy dietary behaviours are main predictors of metabolic impairment later in life^(11,12)^.

Many strategies have been put in place to curb the current trend in childhood obesity, most of which have focused on behavioural changes at the individual level, such as increasing daily exercise and improving food choices, with low rates of success thus far^(13)^. Meanwhile, the lack of nutritional education has been highlighted as a leading promoting factor for unhealthy lifestyles among children^(14)^. According to the Center of Disease Control (2019), nutritional education significantly contributes to improving children’s knowledge and skills to choose healthy foods and beverages^(15)^. The Academy of Nutrition and Dietetics states that the most impactful nutrition interventions in children and adolescents are based on a community-centred, multi-component design including nutrition and physical activity education, school and family involvement^(16)^. This is in accordance with research suggesting that more holistic, community-based, education-focused but also hands-on interventions should be implemented to instil sustainable changes in dietary habits at an early age^(17,18)^. Accordingly, childcare centres and schools are believed to be optimal settings to implement sustainable interventions promoting a healthy lifestyle^(19)^, with positive dietary changes reported particularly in school-age kids^(20–22)^.

Interest in school-based nutrition interventions started after the WHO developed the Nutrition-Friendly School Initiative, its framework was developed from scientific evidence and aims to improve nutrition status of school children. It has five main components within the schools: nutritional policies, awareness and capacity building, nutritional/ health promotion and supportive environment and services^(23)^. The Nutrition-Friendly School Initiative usually includes a committee of parents, teachers, students, health workers and community members^(24)^. This Health Promotion School concept has been adopted in over twenty countries^(25)^.

Several countries in the Arab region have implemented school-based interventions to decrease childhood obesity and overweight. In Lebanon, Jarrib Baleha ('try without it') was piloted in 2010 in two schools. The intervention was successful in increasing nutritional knowledge, increasing intake of water and decreasing intake of soft drinks^(26)^. Another intervention targeted Syrian refugees for a duration of 6 months; it was based on classroom-based education sessions and the provision of locally prepared healthy snacks. Children in the intervention group had increased dietary knowledge, better attitude and improved BMI-for-age^(27)^. In Saudi Arabia, the RASHAKA initiative was a joint school-based intervention between the Ministries of Health and Education and aimed to raise awareness and provide a supportive environment for better nutritional habits and increased physical activity among school children in 4000 schools^(28)^.

Intervention studies having shown positive lifestyle changes have generally combined dietary and physical activity interventions along with improving food choices in school canteens. However, very few have evaluated the role of family involvement on the dietary and physical activity outcomes^(20–22,29)^. In the Middle East, and to our knowledge, the only intervention study assessing the impact of a multi-component nutrition and lifestyle school intervention is the pilot trial conducted prior to the present study^(30)^. Given the need to introduce evidence-based strategies to reduce childhood obesity in the region, the efficacy of global school interventions on dietary habits and the lack of data on such interventions in the Middle East, a randomised intervention was designed combining three components: (1) nutrition and lifestyle education in the school environment, (2) improvements in food quality offered on school premises and (3) parental involvement in the programme. The primary aim of the study was to measure and evaluate the effect of this intervention in five countries on nutrition and physical activity habits in children. The corresponding outcomes were intakes of healthy and unhealthy foods, frequent physical activity, knowledge and self-efficacy scores at baseline and post-intervention. The secondary aim of the study was to evaluate programme implementation in each country using qualitative methodology.

## Methods

### Study design

The study was a one-year intervention in public schools of five different countries – Lebanon, Jordan, Palestine, Saudi Arabia and Bahrain – and included children 9–11 years attending grades 4 and 5. The year of implementation is presented in Table 1. Designed as a cluster-randomised controlled trial, the study included schools selected by Ministries of Health and Education within their jurisdiction. Schools were enrolled in the programme on the basis of their ability to conduct the intervention as per protocol, be it in terms of staff or facilities. Included schools (clusters) were randomised by investigators into intervention and control schools within each country. A total of ninety-one schools were included in the study (forty-five intervention and forty-six control schools), 12 252 students included in the analysis. The minimal required number of schools to be included in the study was set at ten per country based on the results of the pilot study in Lebanon^(30,31)^. Schools were enrolled after submitting consent and assent forms signed by the students, their parents or guardians. All students in grades 5 and 6 were enrolled in the study, and there were no parental refusals. Ethical approval of the study protocol was granted by the Institutional Review Board of the American University of Beirut (AUB) in Lebanon. Additional approvals were obtained by the Ministries of Health and/or Education in Jordan, Palestine, Bahrain and Saudi Arabia.

**Table 1:** Baseline characteristics of the study population

	Control					Intervention				
Country-Year of implementation (number of students)	Number of students (*n* 6200)					Number of students (*n* 6052)				Mean age (years)
	Boys		Girls			Boys		Girls		
	*n*	%	*n*	%	Mean age (years)	*n*	%	*n*	%	
All countries (*n* 12 252)	2455	39·6 %	3745	60·4 %	10·5	2628	43·4 %	3424	56·6 %	10·7
Bahrain-2018 (*n* 3352)	681	37·6 %	1128	62·4 %	10·1	760	49·3 %	783	50·7 %	10·1
Jordan-2015 (*n* 2179)	483	47·3 %	542	52·9 %	10·5	528	45·8 %	626	54·2 %	10·6
Saudi Arabia-2014 (*n* 3129)	760	54·2 %	642	45·8 %	11·4	807	46·7 %	920	53·3 %	11·4
Lebanon-2012 (*n* 1230)	237	44·5 %	295	55·5 %	11·4	302	43·3 %	396	56·7 %	11·4
Palestine-2016 (*n* 2362)	294	20·5 %	1138	79·5 %	9·9	231	24·8 %	699	75·2 %	9·8

### Intervention

Five countries were first selected for this intervention to represent the two main cultural and dietary facets of the region: the Levant (Lebanon, Jordan and Palestine) and the gulf (Saudi Arabia and Bahrain). The programme was coined ‘Ajyal Salima’ and aimed to subsequently be rolled out to other countries in the region. Ajyal Salima was implemented by Health and Education ministries in each country in collaboration with school administration and teaching staff. Students in intervention schools received the programme components over the duration of the school year. In parallel, students enrolled in control schools followed their usual curriculum which did not include nutritional awareness activities. The programme focused on promoting healthy eating and an active lifestyle. Its focus areas included increasing fruit and vegetable consumption, having breakfast daily, minimising the intake of energy-dense foods and beverages and engaging in regular physical activity. The intervention was based on the constructs of the social cognitive theory,^(20,29,32)^ which uses a multilevel approach involving individual changes and environment modification to support positive behavioural changes. Accordingly, the intervention comprised three components, previously detailed elsewhere^(33)^: (1) the classroom component including twelve culturally appropriate classroom interactive sessions addressing nutrition and lifestyle behaviours, (2) the family component consisting of meetings, health fairs and information packets helping families create a supportive environment at home and encourage healthy lifestyle behaviours and (3) the food service component targeting school shops and home-prepared lunchboxes. The implementation of the three components in a coordinated way ensured that the intervention simultaneously targeted knowledge and self-efficacy at the individual level, role modelling at the family level and healthy food access in the school environment. In addition, systematic training of teaching staff delivering the programme was done as part of the fourth component called ‘train of the trainer’. Briefly, this component consisted of train of the trainer workshops, face-to-face teacher training sessions and hands-on coaching on educational activities. Teachers received a complete toolkit to ensure the delivery of the intervention as designed. Although all five countries implemented the same intervention protocol^(33)^, minor country-specific adaptations were made.

### Quantitative data collection and analysis

#### Data collection

Grades 4 and 5 students (aged 9–11 years) in intervention and control schools underwent a baseline assessment 1 week prior to intervention start, and the same assessment was repeated one week following the end of the intervention. Students were asked to fill out a multi-component questionnaire^(33)^ assessing dietary behaviours (thirteen questions), physical activity (ten questions), nutrition knowledge (fourteen questions) and self-efficacy (nine questions). Questions on dietary and physical activity were analysed individually, while answers to self-efficacy and knowledge questions were compounded, and a score was calculated for each outcome. The main outcomes analysed to evaluate the impact of the intervention and reported here are (1) daily intake of breakfast, (2) frequency of healthy and unhealthy foods consumption during the day, (3) frequency of healthy and unhealthy snack consumption between meals, (4) healthy and unhealthy foods bought from the school shop, (5) frequency of eating out/ordering, (6) frequency of physical activity, (7) knowledge score and (8) self-efficacy score. The questionnaire also included four questions assessing parents’ encouragement on healthy eating habits, physical activity and availability of healthy food. The knowledge and self-efficacy questions were summed up to generate a single score (the range for knowledge score: 0–14 – for self-efficacy score: 0–18). The internal consistency (and item-total correlations) of each set of knowledge and self-efficacy items was checked prior to creation of the overall scores.

#### Data analysis

Each outcome was defined based on the corresponding question(s) in the questionnaire. Hence, the healthy food category was defined as fruits and vegetables; unhealthy foods as chips and sugar sweetened beverages (SSB); healthy snacks included fruits while unhealthy snacks included chocolate, cookies, candies, cupcakes, chips, SSB. As for physical activity, the question analysed was ‘do you play or practice activities after school or during weekends, and frequency was recorded as less or more than twice per week’.

Categorical variables with multiple categories were recoded as binary when the outcome of interest was adhering to a specific guideline (consuming breakfast daily = 1, other = 0) or in cases of limited observations in certain categories (never consuming chips grouped with less than twice per day = 0). For nutrition knowledge and self-efficacy questions, the computed score from each set of questions reflected overall levels of knowledge and self-efficacy, respectively.

Descriptive data at baseline and post-intervention was reported as percentage of children having specific dietary and physical activity behaviours (categorical variables), while means and standard deviations were reported for knowledge and self-efficacy scores. Independent sample t-tests were used to compare intervention and control groups on continuous variables and Pearson’s *χ* ^2^ was used to compare intervention and control groups on categorical variables. Binominal logistic regression models were used to estimate the effect of the intervention on binary measures while controlling for gender, countries and baseline measures. Multiple linear regression models were used to estimate the effect of the intervention on continuous variables. Analyses were conducted using Generalised Estimated equations to account for clustering. The Statistical Package for Social Sciences (SPSS) (version 25·0·0, IBM) was used to run all quantitative analyses.

### Qualitative data collection and analysis

Upon completion of the programme, post-intervention qualitative evaluation among programme coordinators was conducted to assess strengths and limitations of intervention components and to identify success factors of the intervention as implemented by each country. All interviews were guided by principal investigators and were intentionally designed to be conversational and open. A set of core questions were used to collect information about the country-specific implementation process. The core questions were followed by a survey that gathered feedback on the strengths and limitations of the programme as implemented by each country. All surveys were filled by programme coordinators and staff members appointed by the ministry of health in each country. All interviews were carried out in English, audiotaped following the programme coordinator’s permission, transcribed verbatim in English and analysed using thematic content analysis, a method described by Burnard^(34)^, which allows to reduce the data into themes.

## Results

### Dietary habits at baseline and post-intervention

**Fig. 1:**
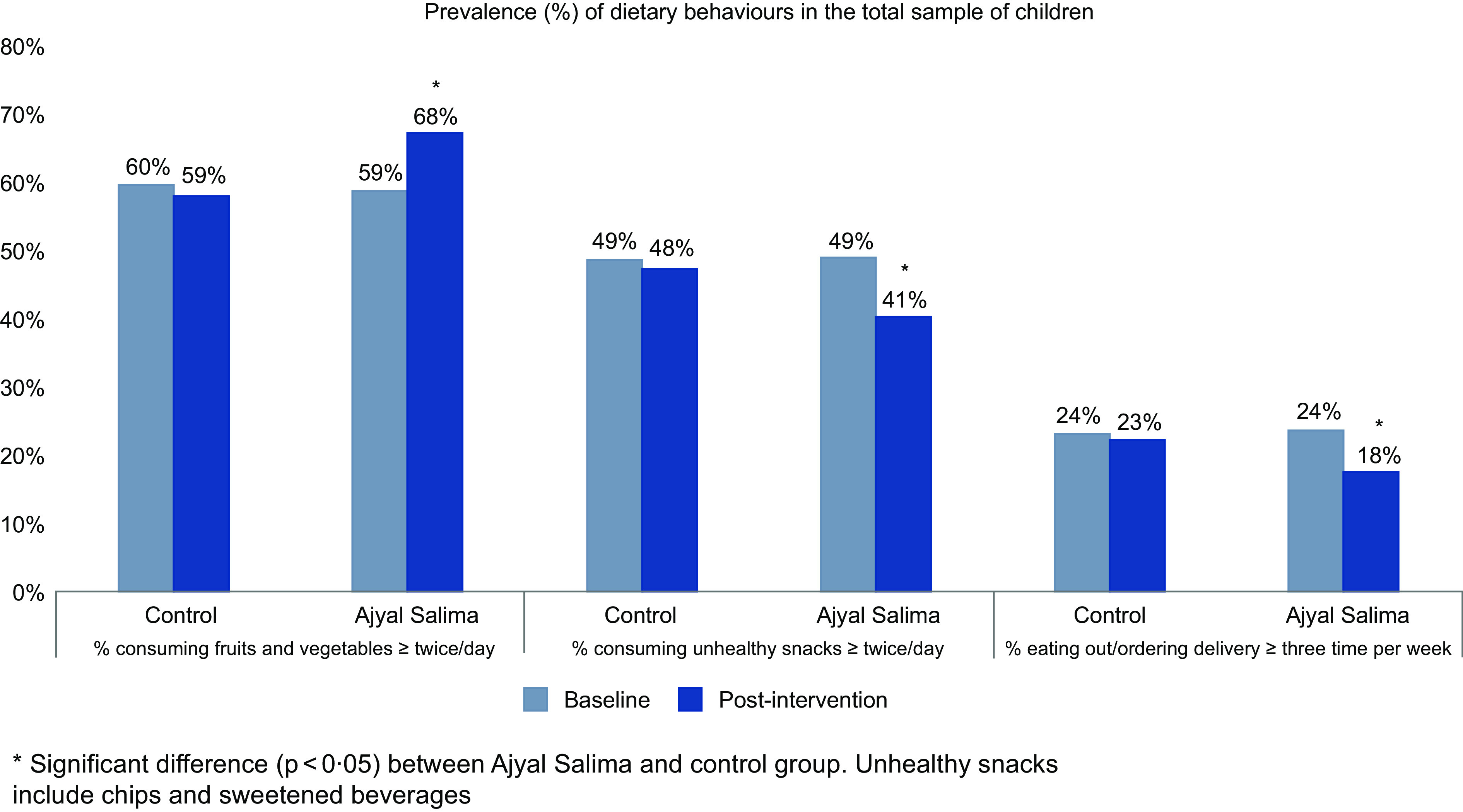
Prevalence of dietary behaviours at baseline and post-intervention

There was no difference between the intervention and control groups at baseline (*P* value > 0·05) in terms of intake of healthy and unhealthy food and eating out. The prevalence of specific dietary behaviours in the total sample at baseline and post-intervention is presented in Fig. 1 showing a significant difference in the proportion of children consuming fruits and vegetables at least twice a day between the Ajyal Salima (70 %) and control (60 %) groups. Similarly, a smaller proportion (41 %) of children was consuming unhealthy foods such as chips and SSB, in the Ajyal Salima group compared with control (48 %). Finally, in the Ajyal Salima group, the percentage of children eating out/ordering in decreased from 24 % to 18 % while this number was maintained at 23 % in the control group post-intervention.

#### Adjusted OR for dietary behaviours in the total sample

Adjusted OR comparing intervention and control groups, adjusted for baseline, were calculated for specific dietary behaviours and presented in Table 2. In the total population, Ajyal Salima intervention students were more likely to improve their consumption patterns than control students. The intervention group had 1·6 times higher odds of having breakfast every day than the control group. Similarly, the intake of healthy foods during the day was likely to be higher and unhealthy foods lower post-intervention as compared to control. A lower OR of eating out or ordering in was also noted for the Ajyal Salima group. While the consumption of healthy snacks was only marginally affected, a reduction in the likelihood of consuming unhealthy snacks was noted post-intervention.

**Table 2:** OR comparing intervention and control groups on dietary habits at baseline and post-intervention in the five study countries

Indicators	All five countries		Bahrain		Jordan		Saudi Arabia		Lebanon		Palestine	
	OR	95 % CI	OR	95 % CI	OR	95 % CI	OR	95 % CI	OR	95 % CI	OR	95 % CI
Breakfast intake everyday (daily *v*. sometimes/never)	1·60**	1·35, 1·90	1·44**	1·11, 1·88	1·29	0·97, 1·72	1·96**	1·28, 3·00	2·04**	1·34, 3·10	1·94**	1·24, 3·04
Intake of healthy food (vegetables, fruits)	1·60**	1·39, 1·84	1·90**	1·55, 2·32	1·24	0·95, 1·62	2·12**	1·40, 3·22	1·16	0·81, 1·67	1·69**	1·22, 2·34
Intake of fruits ≥ 2 times/day	1·69**	1·47, 1·95	1·80**	1·48, 2·20	1·44**	1·16, 1·79	2·33**	1·44, 3·76	1·27	0·86, 1·84	2·01**	1·57, 2·57
Intake of vegetables ≥ 2 times/d	1·46**	1·22, 1·74	1·57**	1·26, 1·96	0·97	0·84, 1·13	2·01**	1·34, 3·02	1·16	0·79, 1·70	1·49**	1·11, 2·00
Intake of unhealthy food (chips, SSB)	0·70**	0·60, 0·81	0·77*	0·63, 0·94	1·12	0·90, 1·42	0·55**	0·39, 0·79	0·46**	0·29, 0·72	0·63**	0·49, 0·80
Intake of chips ≥ 2 times/d	0·70**	0·59, 0·83	0·68*	0·51, 0·91	0·98	0·76, 1·27	0·62*	0·43, 0·89	0·46**	0·29, 0·71	0·62**	0·48, 0·80
Snacks consumed between meals												
Healthy snack (Fruits)	1·27	0·98, 1·65	1·12	0·83, 1·51	1·47	0·95, 2·27	5·63**	2·77, 11·46	0·69	0·38, 1·25	1·85**	1·32, 2·59
Unhealthy snacks (chips, chocolate, cookies, candies, cupcakes and SSB)	0·72*	0·55, 0·96	0·75	0·53, 1·07	0·88	0·42, 1·86	0·38	0·13, 1·05	0·29**	0·13, 0·63	0·64*	0·46, 0·91
Chips	0·74*	0·54, 1·00	0·75	0·50, 1·10	0·87	0·36, 2·07	0·88	0·35, 1·17	0·31**	0·21, 0·47	0·56**	0·39, 0·80
Snacks bought from school shop												
Soft drinks (yes *v*. no)	0·77*	0·61, 0·98	0·58**	0·50, 0·84	0·66	0·16, 2·71	1·84	0·99, 3·43	0·65	0·27, 1·53	0·59*	0·35, 0·99
Chips (yes *v*. no)	0·97	0·75, 1·26	0·74	0·50, 1·11	0·75	0·43, 1·30	–	1·81, 9·12	0·85	0·34, 2·16	0·81	0·49, 1·36
Eating out (three times per week)	0·73**	0·62, 0·86	0·81	0·64, 1·03	0·84	0·61, 1·16	0·52**	0·37, 0·74	0·63	0·39, 1·02	0·65*	0·46, 0·92

* *P* ≤ 0·05.

** *P* ≤ 0·01 showing significantly different OR compared to control.

#### Adjusted odds ratios for dietary behaviours in the five study countries

The effect of the intervention on dietary behaviours varied amongst the five different countries. Adjusted OR as compared with control are reported per country in (Table 2). The likelihood of daily breakfast intake was higher than control in the Ajyal Salima group for all countries except Jordan. In Lebanon, students were twice as likely to have breakfast daily compared with control students.

In the total population, the Ajyal Salima group reported eating more healthy foods during the day than the control group, at the post-intervention assessment. However, the results were not statistically significant in Jordan and Lebanon.

Results also show that the Ajyal Salima group was less likely to consume unhealthy foods such as chips and SSB post-intervention in all countries except for Jordan.

Students from the Ajyal Salima group were more likely to consume healthy snacks between meals after the intervention than those from the control group in Saudi Arabia and Palestine. Notably, the Ajyal Salima group from Saudi Arabia reported 5·63 times higher odds to consume fruits between meals. As for unhealthy snacks including chips, chocolate, cookies, candies, cupcakes and SSB, significant differences with control were seen in Lebanon and Palestine but not in the other countries. In Lebanon, the Ajyal Salima intervention group was 71 % less likely to consume unhealthy snacks than the control group.

Snack choices purchased from the school shop were improved in general as a result of the intervention as Ajyal Salima students in Lebanon, Jordan and Bahrain were less likely to buy SSB and chips than the control group. In Palestine, the reported data show a similar significant decrease; however, numbers reflect snacks bought outside school grounds since a school shop policy prohibiting the sales of SSB and chips was in effect at the time of data collection. As for Saudi Arabia, there was no significant difference between control and intervention schools in terms of SSB purchasing, and the data for purchasing chips were unavailable.

Finally, the likelihood of eating out/ordering in was seen to decrease significantly as compared with control in Saudi Arabia and Palestine only.

**Fig. 2:**
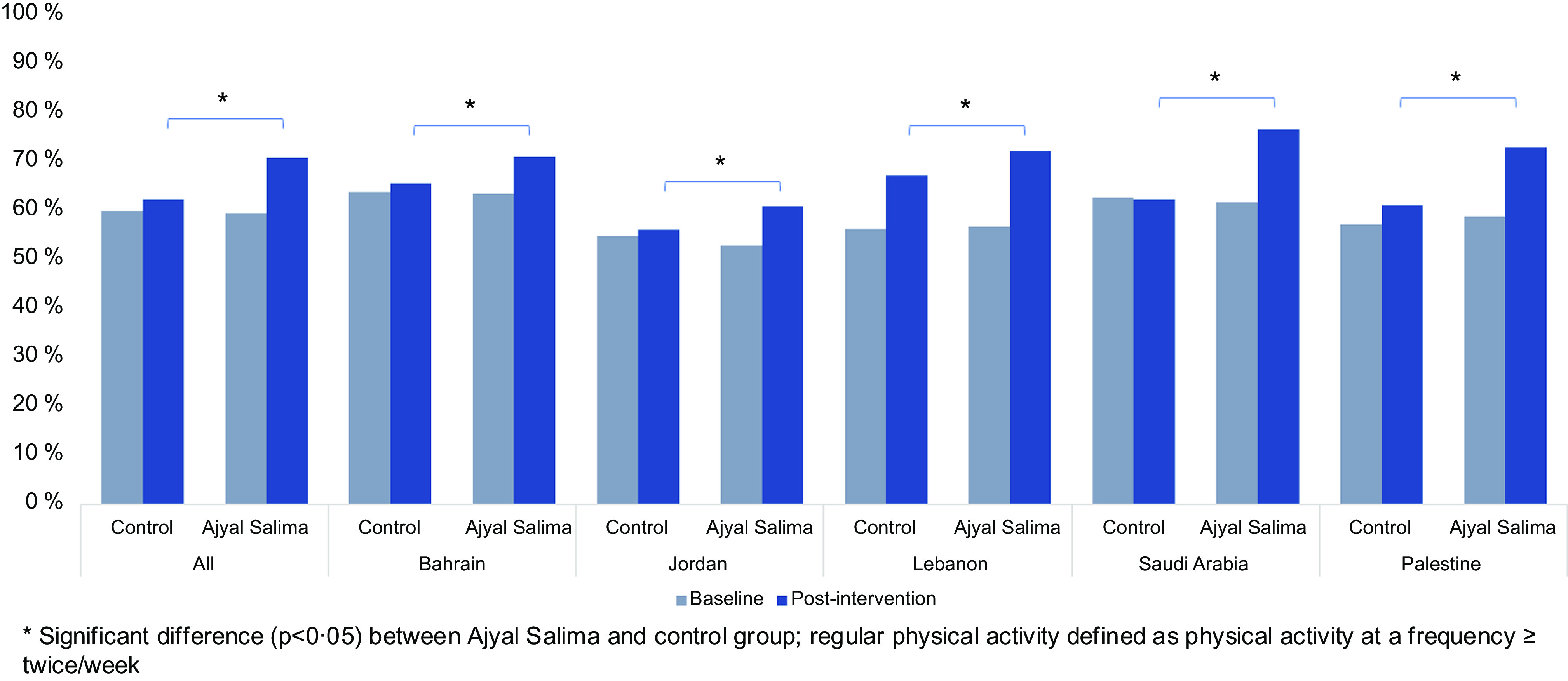
Prevalence of physical activity at baseline and post-intervention

Physical activity at baseline and post-intervention There was no significant difference in baseline values for weekly frequency of physical activities between Ajyal Salima and control students. The percentage of children engaging in regular physical activity after school and on the weekends is presented in Fig. 2 showing a significant difference between intervention and control at the end of the intervention. Indeed, 70 % of children reported engaging in physical activities outside school in the intervention group *v*. 62 % in the control group.

#### Adjusted odds ratios for physical activity in the five study countries

Adjusted odds ratios for engaging in physical activities outside school in the overall sample and per country are presented in Table 3. Overall, the Ajyal Salima group had 1·47 times higher odds to play football, basketball, dance, judo, swimming during weekends or after school than the control students at post-intervention. Differences with control were statistically significant in all countries, except for Lebanon.

**Table 3:** OR comparing intervention and control groups at baseline and post-intervention in the five study countries for physical activities outside school

Indicators	Physical activities outside school ≥ twice/week	
All five countries	OR	1·47**
	95 % CI	1·23, 1·76
Bahrain	OR	1·34*
	95 % CI	1·06, 1·69
Jordan	OR	1·28*
	95 % CI	1·00, 1·64
Saudi Arabia	OR	2·27**
	95 % CI	1·62, 4·17
Lebanon	OR	1·55
	95 % CI	0·86, 2·78
Palestine	OR	1·67**
	95 % CI	1·15, 2·41

* *P* ≤ 0·05.

** *P* ≤ 0·01 showing significantly different OR compared to control.

**Fig. 3:**
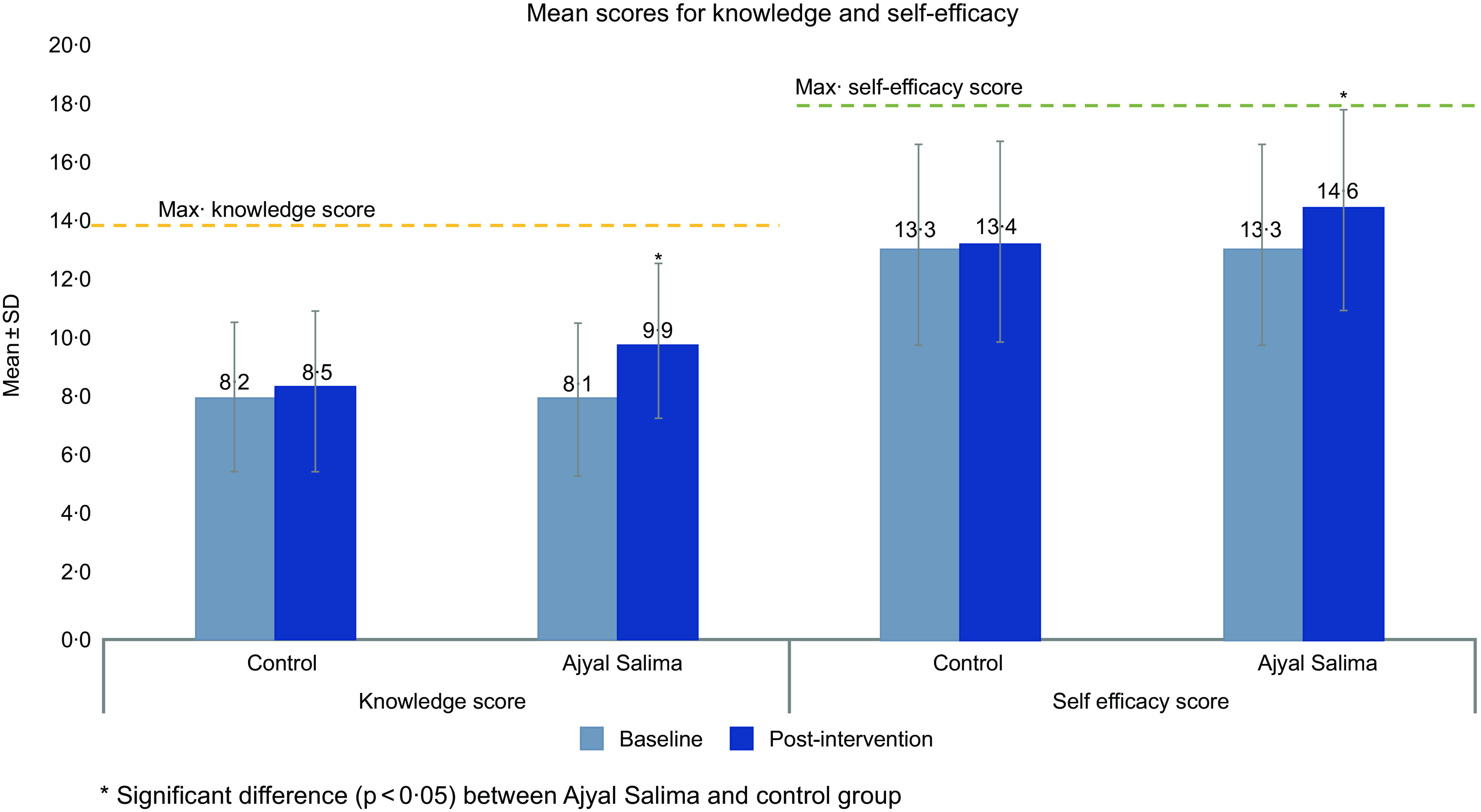
Knowledge and self-efficacy scores at baseline and post-intervention

Knowledge and self-efficacy scores at baseline and post-intervention. Baseline and post-intervention scores were expressed as mean and sd and presented in Fig. 3. There were no significant differences in knowledge or self-efficacy scores between intervention and control groups at baseline. After the intervention, the mean knowledge score was 9·9 ± 2·8 in the Ajyal Salima group, which was significantly different from control students who scored an average of 8·5 ± 2·6. Similarly, self-efficacy post-intervention was higher (*P* < 0·05) in the Ajyal Salima group (14·6 ± 3·3) *v*. the control group (13·4 ± 3·4).

#### Changes in knowledge and self-efficacy in the five study countries

Table 4 shows the coefficients of change in knowledge and self-efficacy scores for Ajyal Salima and control groups. In general, there was a clear improvement in knowledge and self-efficacy as demonstrated by a 1·3 score unit increase in knowledge and a 1·1 score unit increase in self-efficacy when comparing students in intervention group to students in control group after the intervention. The five countries followed the general improving trend for both knowledge and efficacy scores with Palestine reporting the largest change for knowledge (+2·4 score units *v*. control) and Saudi Arabia for self-efficacy (+2 score units *v*. control).

**Table 4:** Knowledge and self-efficacy scores at baseline and post-intervention in the five study countries

Indicators		Knowledge score	Self-efficacy score
All five countries	Coefficient	1·30*	1·08*
	95 % CI	0·88, 1·70	0·72, 1·45
Bahrain	Coefficient	0·91**	0·91**
	95 % CI	0·35, 1·48	0·34, 1·47
Jordan	Coefficient	0·94**	0·68**
	95 % CI	0·46, 1·43	0·14, 1·23
Saudi Arabia	Coefficient	1·95**	2·04**
	95 % CI	1·28, 2·62	1·18, 2·89
Lebanon	Coefficient	1·45**	0·98**
	95 % CI	0·86, 2·03	0·34, 1·62
Palestine	Coefficient	2·43**	1·67**
	95 % CI	1·98, 2·88	1·14, 2·19

* *P* ≤ 0·05.

** *P* ≤ 0·01 showing significantly different OR compared with control.

### Factors influencing children’s dietary, physical activity, knowledge and self-efficacy outcomes

In addition to the main variables analysed in this study, external factors likely to influence children’s behaviour were explored. Results show that gender and parents’ encouragement, as reported by children through questionnaires, modulated dietary and physical activity variables in different study countries.

#### Gender

Behaviours related to dietary habits and physical activity in boys and girls were compared. While there was no overall gender effect on daily breakfast consumption, girls were less likely to consume unhealthy foods than boys post intervention. Physical activity frequency also differed between girls and boys after the intervention, whereby overall, girls were 23 % less likely than boys to engage in physical activities outside school twice or more per week. In terms of knowledge and self-efficacy scores, girls were less likely to improve their knowledge scores at post-intervention than boys in the total population. Meanwhile, changes in self-efficacy scores did not differ between boys and girls.

#### Parents’ encouragement

Most parents (70–75 %) always encouraged their children to exercise and eat vegetables/ fruits while < 10 % of them never encouraged their children to do so post-intervention. Lebanon ranked the highest in terms of parents’ encouragement, whereby parents were the most likely to always encourage their children to exercise or eat healthy food. In the total sample, children whose parents always encourage them to eat vegetables and fruits had 2.16-time higher odds of having healthy food twice or more per week post-intervention than those whose parents sometimes or never did. The same association was found between parents’ encouragement and physical activities, with higher odds of playing sports outside school twice or more per week when parents encouraged children to do so.

### Qualitative results

Overall, data analysis revealed that the intervention programme was culturally appropriate and that educational lessons were fun and engaging. The practical component of the intervention promoted an increase in self-confidence and learning among students and their parents. In addition, positive reinforcement, including positive feedback and small tokens, and parent and teacher engagement were identified as successful components of the intervention. Programme coordinators suggested that more intensive training, changes to the foodservice model, giving teachers more time to prepare lessons, increasing parent engagements, virtual meetings and more physical activity space in the schools were necessary to improve on the intervention. These themes will be discussed in more detail in the paragraphs below and corresponding quotes can be found in the Appendix (Appendix 1).

#### Benefits of the experiential component of intervention

Programme coordinators believed that the visual component used in the intervention programme was beneficial for learning and that the practical component was a good addition to the theoretical concepts. The experiential intervention tools helped students experiment through hands-on activities which helped students connect the theoretical component of the curriculum to daily life and practical applications.

#### Parents and school involvement

Programme coordinators believe that parents need to be more involved in lesson planning and teaching. Also, it was recommended that school principals follow-up on all classroom lessons. During the intervention, some schools struggled to engage parents in the intervention as they had very busy schedules. It was suggested that moving forward, online meetings and digital tools could be offered to increase parent engagement in the programme. Parents were engaged and interested in building the habit of healthy eating in their kids’ lifestyle and many of them adopted healthy habits at home and changed some unhealthy behaviours after attending the introductory meeting and engaged in the parents’ open days. It is believed that engaging parents in the practical component of the intervention increased self-confidence among students. The family section of implementation was easy; however, in some schools, it was hard to keep parents enrolled and engaged due to parents’ motivation and school operations. Accordingly, some schools implemented motivational incentives such as giving parents and students tokens of appreciation and inviting them to take part in practical activities, which promoted a holistic approach to healthy eating mainly on the individual level, the school-level and at home.

#### Training

The intervention training, delivered through ‘Training of Trainers (ToT)’ sessions, received positive feedback; however, participants believed that more intensive training was needed.

#### Teachers’ schedule

Teachers have a busy schedule and are overloaded with other health programmes to implement during the year which made the implementation of this intervention less efficient.

#### Budget constraints

Some schools experienced budget constraints affecting the availability of healthy foods or the infrastructure and equipment for physical activity. Budget constraints were thus a hindering factor to the experiential aspect of the intervention in some schools.

#### School shops

School shops were not always on board and did not all adhere to the healthy eating standards suggested by the intervention programme.

## Discussion

The Ajyal Salima intervention is the first of its kind to be implemented in the Eastern Mediterranean region. Indeed, efficacy data on nutrition education programmes are very scarce in this region, with the only other intervention study^(30)^ having used a multidisciplinary approach being the Ajyal Salima pilot study in Lebanon^(30)^. Our findings show that the multicomponent, school-based nutrition education programme can positively change dietary and physical activity behaviours and improve knowledge and self-efficacy scores in school children 9–11 years of age. Our study population, comprising students from the levant and gulf countries, is representative of the Middle East region which facilitates the generalisability of these results to other countries in the region. Overall, compared to control students, the Ajyal Salima students had higher odds of consuming breakfast daily, consuming healthy foods and exercising regularly outside school hours. This is in agreement with previous research showing that school interventions are most successful when implemented via a holistic approach^(17,18)^ involving parents, teachers as well as the school environment (school shops, school playgrounds and gymnasia). The pan-European study IDEFICS (Identification and prevention of dietary and lifestyle-induced health effects in children and infants) is a good example of a multicomponent holistic school intervention designed according to lessons learned from research on lifestyle modification interventions^(35)^. Unfortunately, and despite a strong study design, the intervention failed to show any effect on children’s behaviours after a 2-year implementation. Nevertheless, lessons learned from IDEFICS can help us understand our findings and improve on future study designs. A long intervention duration, behaviour reporting by parents instead of children and limited parents’ involvement were identified as limitations by the authors^(35)^. Accordingly, the limited duration, narrow age range and older population of Ajyal Salima may have mitigated the variability linked to time, wide age groups and allowed for direct data collection from children. As for parent involvement, it is discussed in detail below.

Although our intervention was successful overall, there were differences amongst countries in the impact of the programme on dietary and physical activity behaviours, while knowledge and self-efficacy were improved across the board. Saudi Arabia and Palestine were the countries where the intervention had a strong impact on every aspect of dietary and physical activity behaviour. Amongst driving factors, identified through the qualitative analysis, are motivational incentives such as rewarding and recognising students and teachers, and actively involving parents in the programme, both particularly observed in Palestine.

It is challenging to pinpoint unique success factors in the current study as the effect of each component was not tested independently and the reason for success is most likely multifaceted. However, by investigating how each country implemented the programme and gathering qualitative data from the feedback of programme coordinators, specific themes were extracted which could partly explain the success of the intervention, as well as the differences in results across the five study countries. First, the experiential or hands-on nature of the intervention was mentioned as the driving force behind the students’ motivation and eagerness to learn nutritional and physical activity concepts. In 2017, DeCosta et al have highlighted the importance of hands-on experience such as gardening and cooking programmes in increasing healthy food consumption in similar educational interventions^(18)^. Second, family involvement was a dominating theme in the qualitative analysis as most country programme coordinators emphasised its contribution to the success of their programme or listed the lack of involvement as a limitation to the implementation of several aspects of the intervention. Some differences observed between countries may be explained by a difference in involvement given that our results show a significant effect of parent encouragement on dietary and physical activity behaviours. In a country like Palestine where the odds of improving all behaviours after intervention were significantly higher than control, we observe that parental involvement was rated high with 70–80 % attendance to parents’ meetings. On the other hand, Jordan where the Ajyal Salima students did not show a significant difference with control on most dietary behaviours post-intervention, recorded 35 % of parents’ attendance to meetings. Parental involvement was highlighted by Summerbell et al in the 2012 paper on recommendations for lifestyle-modification interventions where the authors state that primary prevention is more likely to be successful if parents are involved in the intervention^(36)^. The study by Said et al agrees with this recommendation by showing a strong impact of the parents in modulating dietary behaviours of their children and adolescents^(37)^. In the present study, parent involvement varied in the different countries and depended on the effort the team put in to engage parents and the parents’ motivation to participate. A more direct approach, as defined by Hingle et al would be to request parents’ presence in nutrition education sessions and attendance to family behaviour counseling or parent training sessions. Although proven to be more efficacious than indirect methods such as inviting parents without commitment, this approach may prove hard to implement in certain cultures. In the context of Ajyal Salima, discussions with staff and coordinators captured in the theme ‘family involvement’ revealed suggestions to increase parental engagement including the implementation of a digital platform to invite, inform and engage parents in the programme. Given current trends where most health-related information is sought after online, children and parents could benefit from a platform where they can learn in a fun way and interact with other users in the context of their school programme. Digitalisation could thus bring healthy habits into the household, creating a supportive environment for the child’s learning and making these habits more sustainable.

Finally, for students to apply the knowledge acquired through the educational part of the programme, access to healthy foods and activities in the school environment is fundamental but unfortunately, not always optimal. The extracted theme of budget constraints was especially present in the feedback of programme coordinators in Lebanon and Palestine and encompasses availability of fruits and vegetables on school grounds and accessibility to areas for physical activity. This could partly explain the lack of effect on physical activity behaviours in Lebanon despite the high rate of parents’ encouragement to engage in physical activity. Budget constraints and accessibility to extracurricular activities should be explored as an improvement opportunity reported in our qualitative data (Appendix). In their systematic review of interventions targeting childhood obesity prevention, Brown et al conclude that interventions targeting individual behaviour to prevent obesity are moderately successful, also suggesting that having upstream targets such as policymakers and school management would lead to a greater impact on obesity^(38)^. In agreement with the WHO^(39)^, the authors also state that obesity prevention requires a systems approach where health policy is developed and systematically applied to all sectors in pertaining to children’s education. In the Ajyal Salima intervention, this could be applied in the management of school shops, highlighted as a constraint in some schools where shops are run by owners who are reluctant to make changes at the expense of profit. Targeting school shop policy at a national level and/or providing financial incentives for providing healthy snack options could have a wider impact on children’s dietary habits.

Our study has multiple strengths as well as some limitations. Strengths include the use of a randomised controlled intervention design and the sampling of public schools in different countries that are representative of the region. On the other hand, questionnaire-based methodology can be of concern in terms of internal validity, which is usually threatened by external confounding factors. For this reason, we have used random assignment of schools into control and intervention groups to minimise the effect of potential confounders such as social desirability bias, year of study and differences in implementation between countries. In addition, the Ajyal Salima study purposely used questionnaires and not interview assessments, shown to be less influenced by social desirability in children (40). Another limitation of the study is the different years of implementation for study countries. The Ajyal Salima project required the buy-in from ministries of each country, from the pilot country Lebanon to the rest of the region. Countries were rolled into the programme as soon as the ministries’ approvals were secured. This has led to a staggered implementation over 6 years. The presence of control schools matched for year of implementation in each country minimises the confounding effect of time on the main effect of the intervention. In addition, all statistical analyses were adjusted per ‘country’ to control for country-specific confounders such as year of implementation.

Findings from this study show that the Ajyal Salima intervention, a school-based multi-component educational intervention, leads to significant improvements in dietary and physical activity habits when rolled out in five Middle Eastern countries. These changes are likely to occur via an increase in knowledge and self-efficacy also shown to improve as a result of the intervention. Countries were not equally successful at implementing the intervention or observing behavioural changes; however, modulating factors were identified which could be tackled to enhance the efficacy of the intervention. These factors were parent involvement, accessibility to healthy foods and activity on school grounds and the hands-on aspect of the intervention. Based on previous research, ensuring these three factors are optimised is best accomplished by having upstream targets such as public health policy and school board governance. Indeed, since the first implementation of Ajyal Salima, most countries have put in place school policies with nutrition and physical activity standards set by the ministries, encouraging healthy habits at the individual (nutritious choices) and school (canteens and shops) levels. There is also a need to engage families and communities through conveying the right messages in terms of nutrition and health. In this respect, digitalisation of the programme and its communication can help reach the extended network of students for a more sustainable change in behaviour.

## Supplementary material

For supplementary material accompanying this paper visit https://doi.org/10.1017/S1368980023001489. click here to view supplementary material
