# Supplementary material for: A randomised controlled school-based nutritional intervention in five Middle Eastern countries: Ajyal Salima improved students’ dietary and physical activity habits
Source: Public Health Nutr. 2023 Aug 25;26(10):2036–47. doi: 10.1017/S1368980023001489 (PMC10564613; doi:10.1017/S1368980023001489)
Supplement: Supplementary file 1 [file S1368980023001489sup001.pdf]

: Quotes extracted via thematic analysis

Theme	Lebanon	Jordan	Palestine	Bahrain	KSA
**Training**	*"Theory-based intervention culturally relevant Fun and attractive material Integrative, interdisciplinary and easy to implement"* *"Although school teachers were adequately trained on the programme components and delivery, it is possible that a more intensive training is needed to compensate for the lack of a nutritional background and training."*				*"Male teachers were not motivated, teachers were not trained by the team. No direct passing of information"*
**Teacher's Schedule**	*Teachers have a condensed schedule. In addition to their usual curriculum, they have many health programs to implement during the academic year."*	*"Sometimes teachers found it a bit challenging to cover the session, as they have a condensed curriculum to be covered over the academic semester."*	*"Some portions of lesson has some difficulty in implementing because it needed more time especially for grade 4"*		
**Budget Constraints**	*"We learnt from our discussions that the fruits and vegetables sessions were not always experiential due to budget constraints that limited bringing fruits & vegetables to class." "Students' physical activity is affected by external factors such as limited accessibility to extra-curricular activities, be it due to budget constraints (poor infrastructure or the lack of safe and free places for spontaneous physical activity or play) or homework overload"*		*"Unavailability of some foods and fruits because of budget constraints"*		

**Benefits of Experiential Learning**		*"Sessions provision under the three topics enriched both the official curriculum as the program tools made things visually much more understandable for students and gave the program sessions a base to build on, especially that the practical part of the program applied a lot of the concepts that are introduced in the curriculum."*		*"The training program was based on practical activities which improve learning and motivation, also sharing the experience between schools raised a sense of competition and excitement."*<br>*"In Bahrain we utilize all speciality teachers to implement the program (science-sport-math-social classes) an opportunity that could be used by others "*	*"Well-planned hands on lesson plans, and clear instructions, tools, videos"*
**Parents and school Involvement**	*"Parents have busy schedule"*<br>*"It was challenging for some schools to engage parents in the program enrollment"*<br>*"Improvement: Online meetings to make it accessible to all the parents"*	*"Trying to have all parents on board and engage them in the program activities was a bit hard, I would suggest that we put more effort in getting them on board"*<br>*"I think that the family component implementation was fine, however it was a bit challenging for some schools to get parents on board and engage them in the program enrollment "*<br>*"Parents who attended the open days were highly engaged and interested in applying the new health habits in their houses. Some parents were highly engaged as they attended some in- class activities with their kids, and they helped out the teachers in preparing for the practical part of the lessons and took part in helping the students to work around the activity."*	*"It is important to involve parents in some lessons, school principals need to follow up on all class lessons and evaluate teachers"*<br>*"participation for some parents in class lessons and other extracurricular activities"*	*"Participation of families in the practical activities and attendance of parents help in strengthening confidence of the students"*<br>*"Adding the practical activities of students to the list of task done with parents also involved, this helped ensure participation of parents and making the program more clear to parents to facilitate and ensure motivation of parents and indirectly they will also motivate their children"*<br>*"Inviting parents and focusing on continuous participation was of great addition which facilitate implementation, and spread the knowledge and the culture of having healthy lifestyle in family in addition to the school"*	*"Parents had low attendance and involvement in the intervention"*
**School shops**	*"Some of the school shop owners expressed opposing attitudes towards changing the food items in their shop. .They were concerned about losing profit and were not*	*"Schools' canteens' were not compliant to the healthy standards requested or recommended by the program, thus we can not tell a student to decrease chips consumption while we are still offering*	*Some of the school canteens still need maintenance and rehabilitation, some school canteens need some equipment*		

	willing to prepare healthy food items. " (	*many kinds of chips inside the canteen and not providing other healthy alternatives."*<br><br>*"Turning the canteens control from the teachers' management to a local private provider from the community may help in controlling and forcing a healthy canteen inside the school."*	*(popcorn machine, refrigerator....) to prepare healthy food."*<br><br>*"the policy of school canteens that give the priority to women center to lease out/ rent school canteen support offering healthy alternatives snacks on the other hand , women center is invited to attend ajyal salima training workshop women center receive other training workshop on food processing and food safety MOE develop a guide book titled "*		
